# The variable number of tandem repeats element in *DAT1 *regulates *in vitro *dopamine transporter density

**DOI:** 10.1186/1471-2156-6-55

**Published:** 2005-11-27

**Authors:** Sidney H VanNess, Michael J Owens, Clinton D Kilts

**Affiliations:** 1 Laboratory of Biological Psychopathology, Department of Psychiatry and Behavioral Sciences, Emory University School of Medicine, Atlanta, Georgia, USA; 2 Laboratory of Neuropsychopharmacology, Department of Psychiatry and Behavioral Sciences, Emory University School of Medicine, Atlanta, Georgia, USA

## Abstract

**Background:**

A 40-bp variable number of tandem repeats (VNTR) polymorphism exists in the 15^th ^exon of *DAT1*, the gene encoding the human dopamine transporter (DAT). Though the VNTR resides in a region encoding the 3' untranslated region (UTR) and does not alter the protein's amino acid sequence, the prevalent 10-repeat variant has shown both linkage and association to Attention Deficit Hyperactivity Disorder (ADHD). In this study, we examined the effects of the *DAT1 *VNTR on measures of *in vitro *DAT expression and pharmacology. A series of four *DAT1 *constructs, each containing the *DAT1 *coding region, but varying with respect to the downstream presence or content of the 3'UTR, were engineered and stably transfected into an HEK-293 variant using Flp-In integration, an enzyme-mediated, site-specific recombination technology.

**Results:**

[^3^H] Win 35,428 saturation binding assays and DAT immunoblots revealed statistically significant differences in DAT expression attributable to *DAT1 *genotype. Cells harboring the 10-repeat *DAT1 *variant were characterized by a B_max _approximately 50% greater than cells with the 9-repeat VNTR; those containing only the *DAT1 *coding region or the coding region flanked by a truncated 3' UTR resulted in greater DAT density than either of the naturalistic 9- and 10-repeat variants. Competition binding assays showed no statistically significant *DAT1 *genotype effects on the DAT affinity for methylphenidate, a finding consistent with the positional location of the VNTR.

**Conclusion:**

This study identified the *DAT1 *VNTR as a functional polymorphism and provides an interpretive framework for its association with behavioral phenotypes.

## Background

The human dopamine transporter (hDAT), one member of a family of Na^+^/Cl^- ^dependent transmembrane transport proteins, serves as a critical regulator of dopaminergic neurotransmission throughout much of the brain. The ~64 kb *DAT1 *gene (*SLC6A3*) that encodes the DAT includes 15 exons separated by 14 introns [1]. Concurrent with the cloning and chromosomal mapping of *DAT1 *to the short arm of chromosome 5 [2], a VNTR polymorphism was identified in the 15^th ^exon, a region encoding the transcript's 3' UTR [3,4]. The 40-bp VNTR element is repeated between 3–13 times and in most human populations occurs with greatest frequency in the 9- and 10-repeat forms [5-7]. This polymorphic variation may be evolutionarily young, as a VNTR homologue has been observed in humans, chimpanzees, and cynomologus macaques, but not in lower mammals including the rat and mouse [8-10]. Though the VNTR resides in the 3'UTR and therefore does not affect the protein's amino acid sequence, regulatory factors such as mRNA stability and nuclear transport, and protein synthesis are potentially regulated by such variations [11-14].

Given the prominent role of dopamine neurotransmission in normal and abnormal behaviors, the *DAT1 *VNTR became the object of numerous genetic linkage and association studies [15-20], *in vitro *reporter gene experiments [21-26], *in vivo *SPECT molecular imaging studies [27-30], and pharmacogenetic examinations of the well-documented inter-individual variation in the response to treatment with DAT inhibitors [31-34]. Reports of the association of the *DAT1 *VNTR with ADHD have garnered particular attention, with the balance of evidence relating the 10-repeat VNTR to symptoms of the disorder [35]. Despite its high frequency in the general population [7] and an absence of studies addressing possible effects of the VNTR on measures of DAT physiology and pharmacology, the 10-repeat *DAT1 *allele has become generally recognized as a "high risk" allele for ADHD.

The present study utilized radioligand binding and immunoblotting techniques to compare *in vitro *DAT density and ligand affinity across a series of cell lines stably transfected with constructs containing the *DAT1 *coding region flanked downstream by one of four *DAT1 *3'UTR variants. Flp-mediated recombination, one of a family of site-specific recombination technologies, was used to integrate the *DAT1 *constructs into a common, transcriptionally-active region of the genome, eliminating several caveats associated with traditional reporter gene methodologies [36]. Flp recombinase, the enzymatic basis of the system, is a *Saccharomyces cerevisiae *derived enzyme that utilizes a substrate Flp recombination target (FRT) sequence upstream of a gene of interest (GOI) to site-specifically insert the GOI into a target site in a host cell line, thereby providing a well-controlled approach to establishing the *in vitro *functional effect of the *DAT1 *VNTR polymorphism on the DAT [37].

## Results

### DAT binding assays

Results of three independent [^3^H] Win 35,428 saturation binding experiments revealed statistically significant (p < 0.05) differences in B_max _attributable to *DAT1 *VNTR genotype (Figure 2). Membranes from cells stably transfected with the *DAT1 *coding region (hDAT) displayed the highest mean B_max_. Extension of hDAT with the most proximal ~800 bp of the *DAT1 *3'UTR (hDAT Zero) was associated with decreased *in vitro *DAT density compared to the hDAT line. Of the two prevalent *DAT1 *VNTR variants (hDAT 9 and hDAT 10), cells stably transfected with construct harboring the 10-repeat allele displayed a mean B_max _54% greater than those harboring the 9-repeat variant. Statistically significant differences in *K*_*d *_were observed between *DAT1 *variants in saturation binding assays, though the magnitude of the effect was modest and considered physiologically insignificant (Figure 3); goodness of fit (R^2^) exceeded 0.98 for all non-linear regressions. Three independent competition binding experiments also revealed no pharmacologically significant effects of *DAT1 *genotype on methylphenidate's ability to displace [^3^H] Win 35,428 from the DAT (Figure 4). R^2 ^values were ≥ 0.95 for all competition binding curves.

**Figure 1:**
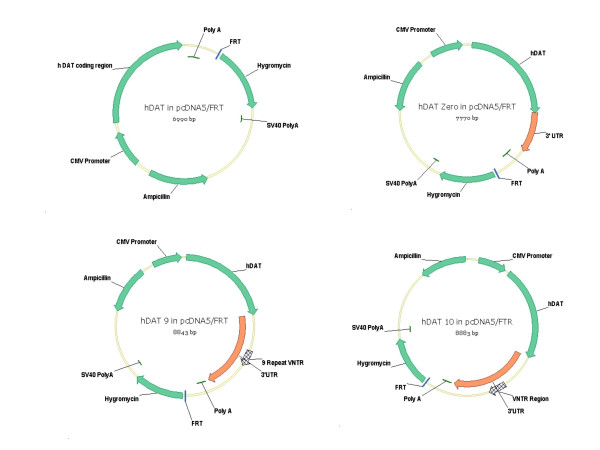
Summary of *DAT1 *constructs used in Flp-In recombination protocol. Top left: **hDAT**, *DAT1 *coding region necessary and sufficient to produce a functional transporter protein. Top right: **hDAT Zero**, a construct containing the *DAT1 *coding region flanked by an ~800 bp fragment of the 3'UTR upstream of the VNTR region. Bottom left: **hDAT 9**, a construct with the *DAT1 *coding region upstream of a full length 3'UTR harboring the 9-repeat VNTR. Bottom right: **hDAT 10**, a construct with the *DAT1* coding region upstream of a full length 3'UTR containing the 10-repeat VNTR.

**Figure 2:**
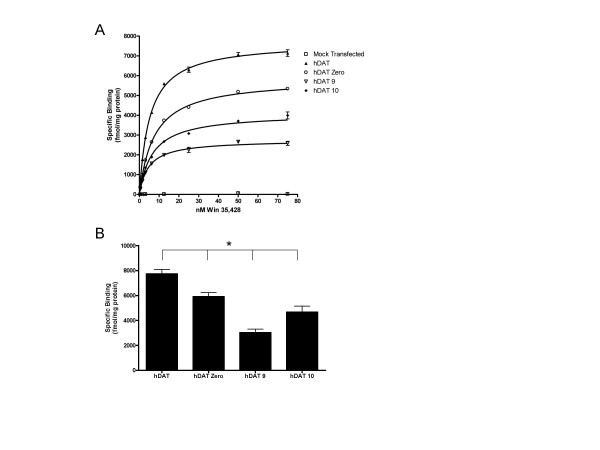
Comparative influence of 3'UTR variation on DAT saturation binding across three independent replicates. **A**) A characteristic binding curve revealing differences in B_max _attributable to *DAT1 *genotype. Mock-transfected negative control cells displayed no specific DAT binding. **B**) A one way ANOVA with Holm-Sidak post-hoc analysis demonstrated genotype-dependent differences in DAT density for all multiple comparisons (* = p < 0.05).

**Figure 3:**
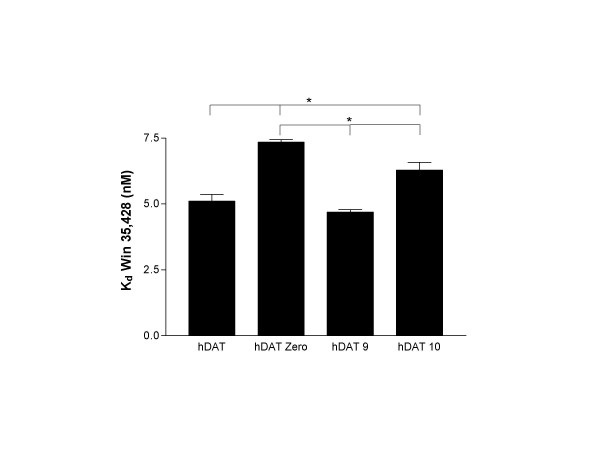
Mean *K*_*d *_measurements derived from three independent [^3^H] Win 35,428 saturation binding assays. A one way ANOVA with Holm-Sidak post-hoc analysis detected VNTR-dependent differences in the DAT ligand affinity (* = p < 0.05) for all but the hDAT-vs-hDAT9 compairson.

**Figure 4:**
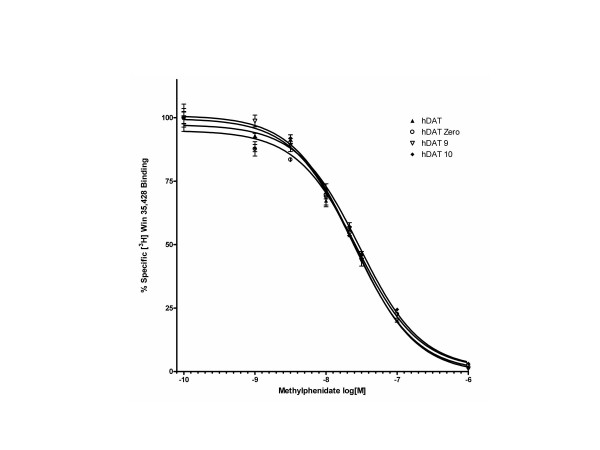
Representative Competition Binding Curves. No pharmacologically significant differences exist between *DAT1 *variants in the encoded protein's affinity for methylphenidate.

### DAT western blots

**Figure 5:**
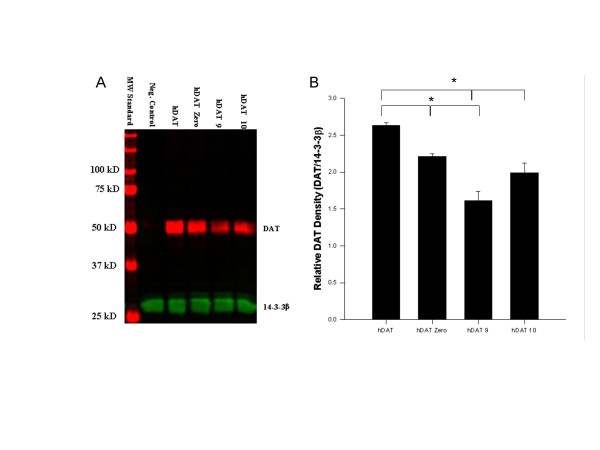
Results of western analysis support the rank order of DAT density observed in saturation binding experiments. **A**) Lane 1: molecular weight marker, Lane 2: negative control, Lane 3: hDAT transfected cell line, Lane 4: hDAT Zero transfected cell line, Lane 5: hDAT 9 transfected cell line, Lane 6: hDAT 10 transfected cell line. Blots were probed both with a rat anti-DAT monoclonal antibody specific for the transporter's N-terminal region as well as a 14-3-3β polyclonal antibody to control for possible protein loading differences. DAT immunoreactivity was detected with an Alexa Fluor 680 conjugated anti-rat IgG (red) and 14-3-3β immunoreactivity with an IRDye-800 conjugated anti-rabbit IgG (green). **B**) DAT immunoreactivity was normalized to the 14-3-3β signal, densiometrically quantified with respect 14-3-3β content, and analyzed via a one way ANOVA with a Holm-Sidak post-hoc analysis. Statistically significant differences (* = p < 0.05) were detected for all comparisons except the hDAT10-vs-hDAT Zero comparison.

The observed rank order of immunoblotted DAT signal intensity across three independent replicates paralleled that of B_max _values derived from saturation binding experiments for the four different *DAT1 *constructs (Figure 5). Mock transfected cells displayed no DAT immunoreactivity. Uniformity of 14-3-3β immunoreactivity between the different transfected cell lines confirmed near equivalent between-lane protein loading and served as a basis for normalization. Significant differences (p < 0.05) were detected for all comparisons except hDAT 10-vs-hDAT Zero.

## Discussion

The VNTR polymorphism of *DAT1*, though residing outside the gene's coding region, is of interest due to its possible influence on the regulation of dopaminergic neurotransmission, its implication in conferring genetic vulnerability for ADHD, and its putative role in modulating response to treatment with a first line medication, methylphenidate. The VNTR has not, however, been characterized as regards its possible influence on DAT physiology and pharmacology. Accumulating evidence that the 3'UTR influences the nuclear export, polyadenylation, subcellular targeting and rates of transcription and degradation of mRNA [11] supports the possibility that a VNTR polymorphism in this region could exert a regulatory influence on gene function. Observed *DAT1 *VNTR effects on reporter gene expression have provided initial support for an effect of the VNTR on *in vitro *gene expression [21-26], though the results vary widely (see [25] for discussion). Furthermore, heterologous reporter gene methodologies are subject to context-specific effects that may not reflect a sequence's impact on a gene of interest [38]. The present study provides multiple lines of converging *in vitro *evidence for the *DAT1 *VNTR's modulatory effect on DAT expression, thus supporting it as a functional polymorphism that may contribute to the recognized inter-individual differences in DAT density and dopaminergic function.

The hDAT cell line, which lacks a 3'UTR altogether, displayed the highest overall DAT density and immunoreactivity, with a B_max _for [^3^H] Win 35,428 binding roughly 2.5-fold that of the hDAT 9 line. The hDAT Zero cell line had a B_max _for [^3^H] Win 35,428 binding ~25% less than that of the hDAT construct, a result that implicates the first ~800 bp of the *DAT1 *3'UTR in diminishing *DAT1 *transcription, mRNA stability, or translation. The additional extension of the 3'UTR to include either the 9 or 10 repeat variant further attenuated B_max _and DAT immunoreactivity compared to that for the hDAT and hDAT Zero cell lines. These results are consistent with the results of reporter gene assays [13,25] in which constructs containing the VNTR polymorphism were associated with decreased *DAT1 *expression. Incremental attenuation of DAT density by both the ~800 bp fragment and the VNTR region suggests the presence of at least two regulatory regions in the *DAT1 *15^th ^exon, with the differential effects of the 9- and 10-repeat variants possibly due to interactions between a VNTR-specific attenuator and one contained within the ~800 bp fragment.

Comparison of the 9- and 10-repeat variants, the two most frequent allelic variations of *DAT1 *[5,7] revealed that DAT binding site density for the 10-repeat VNTR was elevated approximately 50% over that of the 9-repeat allele, with western analysis confirming this finding. This result indicates that the 3'UTR does not simply mediate a length-dependent reduction in DAT availability, as has been previously suggested [22]. Rather, variation in the number of tandem repeats appears to have distinct modulatory influences on *in vitro *DAT density.

At least five groups have reported *in vitro *reporter gene results examining the relative effects of the *DAT1 *9- and 10-repeat VNTR's influence on luciferase or GFP expression in transiently transfected systems. Fuke and coworkers compared the impact of *DAT1 *3'UTRs containing the 7-, 9- and 10-repeat alleles on luciferase reporter activity [21]. Their results suggested that the 10-repeat allele yielded the greatest reporter gene expression. Additionally, it was found that constructs harboring the VNTR polymorphism (of any variety) resulted in lower reporter gene expression than those with no repeat region; our data largely concurs with these observations. However, Inoue-Murayama and colleagues [22], who assessed the relative luciferase activities associated with the human 9-, 10- and 11-repeat alleles in addition to several non-human primate *DAT1 *VNTRs, reported an inverse relationship between reporter gene activity and repeat number, an observation consistent with possible length-dependent reductions in transfection efficiency, and a finding that differs from our data. Miller and Madras [26] arrived at similar findings (i.e. 9 > 10), though their analysis suggested that an additional SNP located in the 3'UTR may further regulate the VNTR's effects. Lastly, Mill [25], who recently published a well-controlled set of reporter gene analyses using both neuronal and non-neuronal cell lines, found no significant difference in reporter gene activity attributable to VNTR copy number. These discrepant studies underscore the need to examine the *DAT1 *VNTR effects under carefully controlled experimental conditions using the DAT protein itself as the reporter signal.

The veracity of our findings is reinforced by the use of a targeted stable integration protocol that eliminates confounds to construct comparison attributable to variable transfection efficiency or clonal variance, both common pitfalls of transient transfection or conventional non-targeted stable integration approaches. Unlike typical stable integration methods, the enzymatically-mediated Flp-In system employed herein resulted in DAT-expressing cell lines with their respective constructs directionally integrated at a common, transcriptionally-active locus [39-41]. pcDNA5/FRT's hygromycin resistance gene, which was used in the isolation and expansion of cell lines for this study, lacks both a promoter and start codon, thereby preventing selection in instances of random genomic insertion. When enzymatically integrated into the host cell line, however, the gene becomes properly aligned with both a promoter and initiation sequence, permitting expression of the hygromycin resistance gene and *DAT1 *variant from a common site. This targeted stable integration approach eliminates the need to normalize data to account for differential transfection efficiency, a significant confound of most traditional reporter gene methodologies.

The current findings are particularly interesting considering the divergent results of four *in vivo *human [^123^I] β-CIT SPECT studies examining the impact of the *DAT1 *VNTR polymorphism on measures of striatal DAT binding potential [27-30]. The first study, conducted in a mixed population of abstinent alcoholics and healthy controls, found that 10-repeat homozygotes displayed a 22% higher DAT binding density than 9/10 heterozygotes [29]. A similar study conducted in a population of healthy subjects revealed sharply different results, with 10-repeat homozygotes characterized by striatal DAT binding 13.4% lower than subjects carrying a 9-repeat allele [28]; van Dyck [30] recently arrived at similar findings. Martinez [27], also utilizing [^123^I] β-CIT, studied a mixed population of 59 healthy controls and schizophrenics and found no difference in binding potential between 10-repeat homozygotes and carriers of the 9-repeat allele. Though these studies differ widely in their conclusions, a number of factors may have contributed to the varied results. Foremost, subject populations varied from healthy individuals to those with psychiatric diagnoses characterized by probable dopaminergic dysregulation. Second, the use of [^123^I] β-CIT, a high affinity DAT ligand, but one lacking selectivity for the DAT over the serotonin transporter, highlights the need to conduct experiments using more selective ligands, preferably in a PET setting [42,43]. Third, two of these studies focused exclusively on the *DAT1 *VNTR polymorphism and did not consider other polymorphisms capable of modulating [^123^I] β-CIT binding (e.g. SERT promoter VNTR polymorphism known to modulate 5-HT transporter density [44]); given [^123^I] β-CIT's binding profile and the partially overlapping brain regional distribution of DAT and SERT, a portion of the between-study variance may be attributable to variations in genes encoding other monoamine transporters. In light of these limitations, PET imaging with highly selective DAT ligands [45] remains the best *in vivo *approach to assessing whether the observed *in vitro *effects of the *DAT1 *VNTR on DAT expression generalizes to the *in situ *DAT.

Of interest are the small, though statistically significant, effects of 3'UTR variations on DAT affinity for [^3^H] Win 35,428. Given the location of the VNTR polymorphism, genotype-dependent variations in the DAT affinity would not be predicted. Preliminary data from our laboratory suggests that VNTR-dependent rates of DAT protein glycosylation may account for the observed variance in ligand affinity, a hypothesis subject to ongoing testing in our laboratory.

While the present study provides evidence supporting the *DAT1 *VNTR as a functional polymorphism, the strength of this assertion is tempered by the inherent limitations of the experimental approach. First, despite the magnitude and reproducibility of the observed effects in the present *in vitro *study, these effects may not generalize to the *in vivo *condition. The association of the 10-repeat allele with an increase in the amount of *in vitro *DAT protein could be attenuated by *in vivo *counter-regulatory mechanisms including upregulation of lysosomal DAT targeting and modulation in rates of endosome-mediated DAT internalization [46-48]. The current experiments, conducted in membrane homogenates, do not discriminate between DAT proteins functionally inserted into the cell membrane and those contained within cellular vesicles. Secondly, the scope of the present study was limited to the effects of the VNTR region independent of other SNP or haplotype effects that may contribute to variability in dopaminergic function; our experiments, by design, do not reflect the recognized complexity of interactions affecting *DAT1 *expression [17,24]. Third, our experiments utilized the powerful CMV promoter; while the use of strong viral promoters is common in experiments such as this, possible promoter/VNTR interactions have been suggested [26]. Therefore, different effects may be observed if the CMV promoter was substituted with either the native *DAT1 *promoter or a weaker constitutively active sequence. Lastly, our results are limited to the effects of the *DAT1 *VNTR in Flp-In 293 cells, a cell line that may lack certain tissue-specific regulatory factors found in DAT rich tissues. While it would be of interest to study these effects in dopaminergic neurons in combination with Flp-In integration, such an approach would render it difficult to pharmacologically distinguish between native and transfected DAT and would significantly compromise the otherwise high degree of experimental control characteristic of the present experiments.

Though the *DAT1 *3'UTR is lengthier than the transcript's entire coding region, is a likely target for modulating *DAT1 *translation, and has been associated with symptoms of ADHD, there exists the possibility of linkage disequilibrium between the *DAT1 *3'UTR and a separate, yet unidentified, causal genetic element. Still, these findings have interpretive significance to the often-observed association of the 10-repeat *DAT1 *VNTR with ADHD. ADHD is viewed as being related to a deficit in synaptic dopamine subserving task-specific signaling. An increase in DAT expression in 10-allele carriers could result in such a dopamine deficit state, depleting dopamine's ability to task-specifically increase signal to noise ratios in target neurons [49,50] and result in deficits in attention and impulse control.

## Methods

### Cloning of *DAT1 *variants

Four *DAT1 *constructs were created, each containing a common promoter, coding region, and poly (A) tail, but varying with respect to the presence and length of the 3'UTR. All constructs were restriction mapped and sequence verified throughout the length of the insert prior to targeted stable transfection in Flp-In 293 cells. Though the sequence of these clones is nearly indistinguishable from that predicted by public genomic databases, they are not eligible for Genbank submission due to the mixed origin (cDNA and genomic DNA) of the constructs. Since the sequence variance in and around *DAT1 *is complex, we have included as supplemental material to this manuscript the complete annotated sequence of each construct (Additional file 1). The constructs, presented in order of increasing length, are depicted in Figure 1 and were generated as follows:

### hDAT

The pRC/CMV plasmid (Invitrogen, Carlsbad, California, USA) containing the *DAT1 *coding region was obtained (courtesy of Dr. Marc Caron, Duke University) and the insert released via digestion with PmeI (New England Biolabs, Beverly, MA, USA). The resultant fragments were electrophoretically resolved on 1.5% agarose and the band containing the *DAT1 *coding region was purified using the QIAquick Gel Extraction Kit (Qiagen, Venlo, The Netherlands). Similarly, pcDNA5/FRT was digested with PmeI, the vector backbone purified (QIAquick PCR Purification Kit) and treated with shrimp alkaline phosphatase. The *DAT1 *insert underwent blunt end ligation into the pcDNA5/FRT backbone by overnight reaction at 14°C with T4 DNA ligase (New England Biolabs).

### hDAT Zero

An ~800 bp segment of the *DAT1 *3'UTR beginning upstream of the stop codon and terminating proximal to the VNTR element was cloned from banked human DNA into pCR 2.1 TOPO using the TOPO TA Cloning Kit (Invitrogen). Oligos *DAT1*E15F (5'-CAACCACAGTCTCGCGGCTTT-3') and *DAT1*E15ZeroR (5'CTCAGGCCGTTCCCTACACC-3') were used in conjunction with Platinum Pfx DNA Polymerase (Invitrogen) to drive 35 cycles of PCR using the manufacturer's recommended protocol. Following initial amplification, 1 U of Taq polymerase was added to the reaction mixture and incubated at 72°C for 10 minutes; the resultant PCR product with 3' A-overhangs was used immediately in a TOPO-TA cloning reaction. After transforming competent bacteria and identifying clones containing the insert of interest, the fragment containing the 3'UTR was released via restriction digestion with BsmBI and XbaI (New England Biolabs), band purified, and cloned into a BsmBI/XbaI site in the pRC/CMV plasmid immediately downstream of the *DAT1 *coding region. The resultant clone contained an insert consisting of the *DAT1 *coding region flanked by a ~800 bp fragment of the 3'UTR up to, but not including, the VNTR region. This construct was subcloned into the pcDNA5/FRT plasmid via the same blunt end cloning method used in the generation of the hDAT plasmid.

### hDAT9 and hDAT10

The *DAT1 *15^th ^exon from a previously genotyped 9/10 heterozygote was PCR amplified and cloned into pCR2.1-TOPO using a protocol similar to that used in the generation of the hDAT Zero construct. Primers *DAT1*E15F (sequence previously noted) and *DAT1*E15R (5'-AGGGACCCACACGATGCTGA-3') were used to produce a ~2100 bp PCR product that was subsequently made TOPO-TA compatible through the previously described A-overhang procedure. TOPO-TA reactions were immediately performed and used to transform competent bacteria. Ampicillin-resistant bacterial colonies were isolated and grown overnight using standard culture conditions. Plasmid DNA was isolated and genotyped to differentiate those containing the 9-repeat versus the 10-repeat VNTR via a protocol that has been previously described [4]. Following identification of clones containing the 9 and 10 repeat VNTRs, respectively, the insert containing the entire 3'UTR was released via restriction digestion with BsmBI and XbaI. The band was purified via gel extraction and cloned into a BsmBI/XbaI site in the original pRC/CMV plasmid. The resultant clones contained an insert consisting of the *DAT1 *coding region flanked by a ~1900 bp fragment of the 3'UTR harboring either the 9 or 10 repeat variant. The insert was subcloned into the pcDNA5/FRT plasmid via the previously described protocol.

### Creation and maintenance of stably transfected model cell system

The Flp-In 293 host cell line (Invitrogen), an HEK-293 variant containing a Flp-In recombination site at a transcriptionally active locus, was grown in complete medium (D-MEM with 2 mM L-glutamine, 10% FBS, 1% penn/strep) supplemented with 100 μg/mL Zeocin in a 37°C incubator with 5% CO_2_. 48-hours prior to transfection, cells were split into 6-well plates and grown to ~80% confluence on the day of transfection and incubated in complete medium lacking Zeocin. Cells were co-transfected with one of the four *DAT1 *variants and pOG44 (Invitrogen), a plasmid encoding the Flp-recombinase enzyme necessary for targeted stable integration [51]. The Lipofectamine 2000 reagent was used to transfect the cells according to the manufacturer's recommended protocol (Invitrogen). Briefly, 3.6 μg pOG44 and 0.4 μg of one of the four *DAT1 *constructs were diluted to a 250 μl volume in Opti-MEM reduced serum media (Invitrogen); similarly, 10 μL Lipofectamine 2000 was diluted to a volume of 250 μL and incubated at room temperature for 5 minutes. The diluted DNA and Lipofectamine solutions were combined and incubated at room temperature for 20 minutes and then added to culture medium. 24-hours after transfection, cells were washed with PBS and fresh complete medium was again added. 48-hours after transfection, cells were split into fresh medium, plated on 150 mm × 25 mm cell culture dishes, and incubated at 37°C until cells attached. Medium was then removed and replaced with complete medium supplemented with the selecting antibiotic hygromycin (100 μg/mL). Selective media was replaced every 4 days until hygromycin-resistant foci were identified. Single resistant colonies were encircled with a cloning cylinder, dislodged with 0.25% trypsin, and expanded. Cells exhibiting the phenotype for proper Flp-In recombination and demonstrating the presence of a cocaine-sensitive DAT as assessed by a rapid single concentration [^3^H] dopamine uptake assay were selected; clones were expanded to confluence in 2-tray (1264 cm^2^) Nunc Cell Factories (Nalge Nunc International, Rochester, NY, USA). Once confluent, the four cell lines were harvested with 37°C PBS containing 0.53 mmol/L ethylenediaminetetraacetic acid, separated into aliquots, and centrifuged at 2000 × *g *for 10 min. Supernatants were decanted and the pellets were rinsed with 37°C PBS. Pellets were again centrifuged at 2000 × *g *for 10 min, the supernatants decanted, and the pellets stored at -80°C until the time of assay. Mock transfected cells were confirmed to have no specific DAT binding or immunoreactivity prior to initiation of assays.

### General radioligand binding methods

Binding assays were carried out in 0.03 M phosphate buffer (pH 7.4) containing 0.32 M sucrose. Membrane suspensions were prepared by resuspending the pellet in 7 mL of ice cold assay buffer followed by homogenization with a Polytron PT3000 for 20 seconds at 20,000 rpm. All assays were performed in 12 × 75 mm polystyrene tubes in a 1,000 μL final volume consisting of 800 μL of assay buffer, 100 μL of [^3^H] Win 35,428 (Perkin Elmer, Boston, MA), and 100 μL of cell membrane suspension. Competition assays were performed in a final volume of 1,000 μl consisting of 700 μl assay buffer, 100 μL of [^3^H] Win 35,428, 100 μL of methylphenidate HCl (10^-10 ^to 10^-6 ^M) (Sigma-Aldrich, St. Louis, MO), and 100 μl of cell suspension. Nonspecific binding was defined via the addition of 25 μM cocaine HCl (Sigma-Aldrich). Incubations were terminated via rapid vacuum filtration through GF/B filters presoaked in assay buffer containing 0.3% polyethyleneimine and rinsed with three washes (5 mL) of ice cold assay buffer. Filters were punched, placed in scintillation vials, and equilibrated overnight in 6 mL of liquid scintillation cocktail (Ultima Gold, Packard, Meriden, CT, USA). Vials were shaken and radioactivity determined in a liquid scintillation counter at 50% efficiency for 240 sec/vial.

### Saturation binding assays

The DAT radioligand [^3^H] Win 35,428 was isotopically diluted with freshly weighed cold ligand (courtesy of Dr. Michael Kuhar, Emory University) in silanized borosilicate glass tubes. Drug was initially dissolved in assay buffer containing 5 mmol/L HCl at a concentration of 1 μg/μL followed by serial dilution with assay buffer. Saturation binding assays utilized final [^3^H] Win 35,428 concentrations over a range of 0.5 to 75 nM. Total and nonspecific binding was determined in triplicate at each concentration of ligand. Assays were initiated by the addition of membrane suspension and incubated at 4°C for 4 hours.

### Competition binding assays

[^3^H] Win 35,428 was isotopically diluted with freshly weighed cold ligand in silanized borosilicate glass tubes as previously described. The concentration used in competition assays (5.85 nM) approximated the mean *K*_*d *_for [^3^H] Win 35,428 binding to the DAT from the binding site saturation studies. Methylphenidate was dissolved in assay buffer containing 5 mmol/L HCl at an initial concentration of 1 μg/μl. The concentrated solution subsequently underwent serial dilution to generate final concentrations of competing ligand ranging from 0.01 to 10,000 nM. Assays were initiated by the addition of membrane suspension, and incubated at 25°C for 1 hour.

### Protein assays

At the time of each saturation binding assay, twelve 100 μL aliquots of each membrane homogenate were collected for purposes of measuring total mean protein content with a BCA protein assay (Pierce Biotechnology, Rockford, IL, USA). Protein assay results were used in the normalization of saturation binding data to account for minor differences in total added tissue.

### Western blots

Cell pellets were homogenized in PBS in the presence of DNAse and protease inhibitors (Roche Applied Science, Indianapolis, IN, USA). Samples were then enzymatically deglycosylated (Enzymatic Protein Deglycosylation Kit, Sigma-Aldrich) prior to the loading and electrophoretic resolution of 20 μg total protein in sample buffer (40 mM Tris, pH 6.8, 0.1% SDS, 10% glycerol, 0.025% Bromphenol blue) on 10% acrylamide. DAT, a highly glycosylated protein, typically runs as a smear of high molecular weight bands; deglycosylation reduces the core protein to a mass of ~50 kD and greatly aids densitometry analysis. An example of mature, fully-glycosylated DAT and the resultant high molecular weight smear is included as supplemental material (Additional file 2) to demonstrate the significant benefit of sample deglycosylation. BioRad's Precision Plus Protein Standards (Hercules, CA, USA) were loaded into the first lane of each gel. Gels were transferred to PVDF membranes (Millipore, Billerica, MA, USA) overnight at 3 mA. Membranes were blocked for 1 hour at RT (SuperBlock Blocking Buffer, Pierce Biotechnology) on an orbital shaker and probed with a rat anti-DAT monoclonal antibody specific for the protein's N-terminal region (1:250 dilution, courtesy Dr. Allan Levey, Emory University) and rabbit anti 14-3-3β polyclonal antibody (1:500 dilution, Santa Cruz Biotechnology, Santa Cruz, CA, USA) at room temperature for 1 hour. Following rinsing, the blot was incubated with a 1:10,000 dilution of both Alexa Fluor 680 conjugated anti-rat IgG (Molecular Probes, Eugene, OR, USA) and IRDye-800 conjugated Anti-Rabbit IgG (Rockland Immunochemicals, Gilbertsville, PA, USA) for 1 h at room temperature. Blots were rinsed, dried and visualized using a LI-COR Biosciences Odyssey Infrared imager (Lincoln, NE, USA). The ubiquitously expressed 14-3-3β was used as a protein loading control. Western analyses were conducted in triplicate.

### Data analysis

#### Radioligand binding data

Saturation and competition binding curves were analyzed by the iterative, non-linear, curve fitting program Prism 4.0 (GraphPAD Software, Inc., San Diege, CA). Values for *K*_*d*_, B_max _and *K*_*i *_are expressed as mean values ± S.E.M (Table 1). A one-way ANOVA with a Holm-Sidak post-hoc analysis was used to identify statistically significant differences between VNTR variants. The threshold for statistical significance was set at p < 0.05.

**Table 1:** *In vitro *pharmacological characteristics of *DAT1 *variants.

	**hDAT**	**hDAT Zero**	**hDAT 9**	**hDAT 10**
B_max _(± S.E.M)	7747 (± 360)	5913 (± 308)	3021 (± 286)	4669 (± 480)
K_d _(± S.E.M)	5.44 (± 0.55)	7.77 (± 0.41)	4.98 (± 0.20)	6.69 (± 0.57)
K_i _(± S.E.M)	24.77 (± 1.14)	29.13 (± 1.81)	25.00 (± 0.10)	33.39 (± 4.15)

### Western blot analysis

Western blots underwent densitometric analysis using the program ImageJ 1.33 u (National Institutes of Health, Bethesda, Maryland, USA). The DAT signal was normalized to the 14-3-3β signal within each lane (expressed in arbitrary units), then subsequently examined for main effects of *DAT1 *genotype via a one way ANOVA with a Holm-Sidak post hoc comparison. The threshold for statistical significance was set at p < 0.05.

## Authors' contributions

SHV performed all molecular biology, radioligand binding experiments, immunoassays, statistical analysis and drafted the manuscript. MJO participated in the design of the pharmacological aspects of the study, provided supervision of radioligand binding experiments, and aided in the revision of the manuscript. CDK participated in the overall design and coordination of the study and aided in the drafting of the manuscript. All authors read and approved the final manuscript.

## Supplementary Material

Additional File 1 ZIP archive containing full length annotated sequence files (.gb format) of the four *DAT1 *constructs. dat1_clones_in_pcDNA5-FRT.zip (17 K) Click here for file

Additional File 2 Western blot run without sample deglycosylation. Samples are variably glycosylated, creating a high molecular weight smear. Sample deglycosylation yields tight, discrete bands that are more easily analyzed. Click here for file
