# Supplementary figures and images for: The variable number of tandem repeats element in DAT1 regulates in vitro dopamine transporter density
Source: BMC Genet. 2005 Nov 27;6:55. doi: 10.1186/1471-2156-6-55 (PMC1325255; doi:10.1186/1471-2156-6-55)

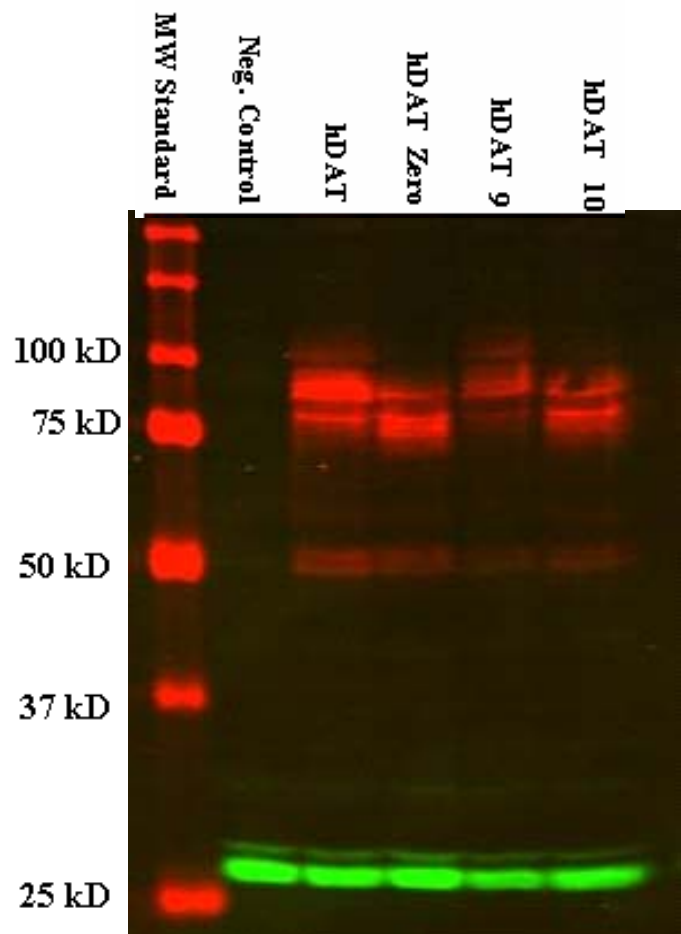

Supplement: Additional File 2 — Western blot run without sample deglycosylation. Samples are variably glycosylated, creating a high molecular weight smear. Sample deglycosylation yields tight, discrete bands that are more easily analyzed. [file 1471-2156-6-55-S2.pdf]
